# The effect of visual support strategies on the quality of life of children with cerebral palsy and cerebral visual impairment/perceptual visual dysfunction in Nigeria: study protocol for a randomized controlled trial

**DOI:** 10.1186/s13063-019-3527-9

**Published:** 2019-07-10

**Authors:** Roseline Duke, Komomo Eyong, Kathryn Burton, David MacLeod, Gordon N. Dutton, Clare Gilbert, Richard Bowman

**Affiliations:** 1 London School of Hygiene and Tropical Medicine, International Centre for Eye Health, London, WC1E 7HT UK; 2 Calabar Childrens’ Eye Center, Department of Ophthalmology, University of Calabar Teaching Hospital, Calabar, Cross River State Nigeria; 3 Paediatric Neurology Unit, Department of Paediatrics, University of Calabar Teaching Hospital, Calabar, Cross River State Nigeria; 4 Cambridge Community Services NHS Trust, Cambridge, UK; 5 Glasgow, UK

**Keywords:** Cerebral palsy, Cerebral visual impairment, Perceptual visual dysfunction, Insight Questions Inventory, Visual support strategies, Paediatric quality of life

## Abstract

**Background:**

Cerebral visual impairment (CVI), including perceptual visual dysfunction (PVD), is common in children with cerebral palsy (CP). Inventories of questions relating to practical aspects of visual perception in everyday life, in particular the closed-ended Insight Questions Inventory (IQI), can be used to assess CVI/PVD. Studies linking responses to the inventory with specific visual support strategies, aimed at modifying the child’s environment and/or behaviour to minimize the impact of the CVI/PVD, have been piloted. The IQI and tailored strategies have not been used in an African population, nor have they been tested in a controlled trial. This trial will compare the effectiveness of the IQI and linked visual support strategies versus general supportive treatments on the quality of life of children with CVI/PVD and CP through a randomized controlled trial.

**Methods/design:**

This is a prospective, double-blind, parallel-arm, randomized controlled trial. The primary outcome is change in quality of life scores between the two arms of the trial at 6 weeks, assessed using the Paediatric Quality of Life Inventory (PedsQL) generic 4.0 and CP 3.0 module. All children will undergo baseline assessment including the Open Questions Inventory, IQI, PedsQL 3.0, PedsQL 4.0 generic, and the Strengths and Difficulties Questionnaire (SDQ). Eligible children with CP aged 4 years to < 16 years will be stratified and blocked by the age groups 4–9 and 10 to < 16 years and by Gross Motor Function Classification System (GMFCS) levels 1–3 and 4–5. Families in the intervention arm will receive tailored insight visual support strategies and telephone calls during the 6-week trial period. The control arm will receive standard treatment and the intervention after the 6-week trial period. Follow-up interviews will be performed in both arms at 6 weeks with a repeat administration of the PedsQL CP 4.0 and 3.0, the IQI and the SDQ. Secondary outcomes include a change in functional vision.

**Discussion:**

This randomized controlled trial will provide evidence of the effectiveness of this intervention for children with CP in a resource-poor setting.

**Trial registration:**

Pan African Clinical Trials Registration, PACTR201612001886396. Registered on 3 December 2016.

**Electronic supplementary material:**

The online version of this article (10.1186/s13063-019-3527-9) contains supplementary material, which is available to authorized users.

## Background

Cerebral palsy (CP) is the most common cause of motor disability in children in low-income countries, affecting the well-being of children and carers [1, 2]. It has a worldwide incidence of about 2–2.5 per 1000 live births [1, 3, 4]. CP describes a group of permanent developmental disorders of movement and posture causing activity limitation, which are attributed to non-progressive disturbances in the developing fetal or infant brain. In most individuals with CP, the motor impairment is accompanied by secondary musculoskeletal problems, epilepsy, disturbances of sensation, perception, cognition, communication, and behaviour [5–7]. The definition of CP highlights that the motor and visual impairments in CP are integral components of the same disorder [5].

The diagnosis of CP is based on a clinical assessment of history and examination [8]. For clinical and research purposes, CP is classified by the nature of the movement disorder (spasticity, ataxia, dystonia, and athetosis), and the anatomic distribution of the motor abnormalities [7]. Current standardized classifications of CP include the Gross Motor Function Classification System (GMFCS), the GMFCS expanded and revised (E&R), the Manual Ability Classification System (MACS), and the Communication Function Classification Scales (CFCS) [9, 10]. There is currently no scale assessing visual or sensory function.

Facility-based studies on children with CP in Africa have shown high rates of the more severe forms (using GMFCS) of CP and associated conditions [11, 12], such as epilepsy, learning disability, deafness, speech disorders, visual impairment and malnutrition [13].

The development of normal vision takes place over the first few years of life as anatomical structures and physiological processes mature [14]. This occurs at the same time as the development of language, emotion, motivation, and mental functions (which include cognition, social functions and the motor systems), which means that poor visual function can affect these functions and vice versa. Behaviour mirrors the cooperation and close integration of these functional systems [5, 15]. Mental and behavioural difficulties can be assessed using the Strengths and Difficulties Questionnaire (SDQ), a screening tool for 3–16 year olds (not specific to CP). It has 25 items on psychological attributes across five domains: emotional symptoms, conduct problems, hyperactivity/inattention, peer relationship and pro-social behaviour [16].

Cerebral visual impairment (CVI), the most common form of visual impairment in CP [17, 18], refers to loss of visual function from lesions in pathways from the lateral geniculate body and/or from the cortex to the higher centres. The latter, known as the perceptual visual system of the brain, includes the dorsal (occipito-parietal) and the ventral stream functions (occipito-temporal) [19, 20]. Lesions between the chiasm and cortex can lead to loss of visual acuity and/or defects in the visual fields. Lesions in the perceptual visual systems give rise to perceptual visual disorders (PVD) such as problems with face recognition, finding objects in a complex visual environment, or orientation in space. These have received little attention compared with loss of acuity or the visual field, but studies have shown that PVD can impact on a child’s ability to function using vision and adversely affects quality of life [21–23]. The deficits in CVI/PVD may be mild or severe, ranging from mild PVD with normal visual acuity and visual fields, through to complete loss of all visual function, depending on the site(s) and severity of the lesion(s). The term CVI/PVD is used to reflect the spectrum of disorders. Children affected by CVI/PVD may also have intellectual and attentional disorders.

As many brain functions are subconscious, such as visual guidance of movement and visual search, CVI/PVD may be asymptomatic or go unrecognised. In addition, in children with CP, motor deficits can mask disordered visual guidance of movement. [23].

CVI/PVD can be suspected from observed visual behaviours, and becomes more apparent with age. The more severe features can be assessed by 4–5 years [24]. Children who do not have sufficient language or who are unaware of their condition cannot report or comment on what they can see or why vision is problematic and, therefore, behaviour-based methods are used for assessment [25–27].

Although formal psychological testing may be helpful in identifying children with CVI/PVD [28], the appropriateness of psychological tests in a clinic setting is limited, and testing may be hindered by visual disorders and other disabilities. The functional impact of CVI/PVD can be identified by careful observation of behaviour, aided by structured history taking using open or closed questions. Positive unprompted answers to non-leading questions (open inventory) can be helpful in eliciting characteristic visual behaviours resulting from severe visual impairment [29–31], visual field defects, and cognitive and perceptual visual disorders, and has been validated. Structured (closed questions) history taking using the Insight Questions Inventory (IQI), a 52-item inventory which has been used in the UK, Bangladesh and India [3, 14, 21, 30, 32], has been shown to be helpful; responses correlate with psychophysical tests of visual perception and quality of life scores. In addition, the responses lead to specific visual support strategies aimed at modifying behaviour and/or the environment to compensate for the visual problems and to minimize their impact. The IQI assesses seven domains of visual function: visual field, perception of movement, visual guidance of movement, visual search, visual attention, recognition and navigation, and behaviour in crowded environments. A simple software program links inventory response to the appropriate visual support strategies so that each child/family has a set of recommended visual support strategies (hereafter called IQI vision support strategies) specific for that particular child. Despite anecdotal evidence that parents find the inventory and strategies useful, there is no clinical trial evidence of the effectiveness of these strategies on improving visual function in everyday life and quality of life compared with standard ocular treatments such as the use of spectacles and strabismus surgery when required.

Although closed questions lend themselves to specific strategies, responses may be subject to cultural bias. In this study, we will compare findings using the open and closed questions. The use of targeted, tailored strategies in the management of CVI is in line with patient-centred care [33]. The parents and caregivers of the child with CP and CVI are part of the decision-making process, and implement the intervention. Several clinical trials to improve the quality of life of children with CP have been undertaken but none have addressed improvement of visual function [34, 35].

The paediatric quality of life literature includes both generic and condition-specific instruments. The Paediatric Quality of Life Inventory (PedsQL) 4.0 generic core scales encompass the following four areas of functioning: physical (eight items); emotional (five items); social (five items); and school (five items). It was used in a similar cross-sectional study in Bangladesh in which 180 children were recruited where 57 (32%) had visual acuity impairment and 95 (53%) had some parent-reported PVD. PVD was reported to be an important contributor in reducing quality of life in children with CP, independent of motor disability and impaired visual acuity [21].

The 35-item PedsQL 3.0 CP module encompasses seven scales: 1) daily activities (nine items); 2) school activities (four items); 3) movement and balance (five items); 4) pain and hurt (four items); 5) fatigue (four items); 6) eating activities (five items); and 7) speech and communication (four items).

### Rationale

The rationale of the study is to test the following hypotheses: 1) structured history taking from parents or caregivers (hereafter referred to as carers) is an effective way of determining whether children with CP have CVI/PVD; and 2) if explained to carers and carefully applied, visual support strategies, tailored to the question inventory responses, can minimize the impact of CVI/PVD on the child’s life and hence improve quality of life and everyday visual function.

## Methods

### Aim

The aim of this study is to improve the management and outcome of CVI/PVD in children aged 4 to less than 16 years of age with CP in a lower- to middle-income country.

### Objectives

The objectives are: 1) to improve the management and outcome of CVI/PVD in children aged 4 to less than 16 years of age with CP through the administration of the IQI visual support strategies; 2) to determine the effect on the quality of life and visual function of children aged 4 to less than 16 years of age with CP following the application of specifically targeted IQI visual support strategies compared with general supportive management in Cross River State, Nigeria; and 3) to determine whether the degree of motor or visual dysfunction is associated with the degree of improvement in quality of life.

### Trial design

**Fig. 1:**
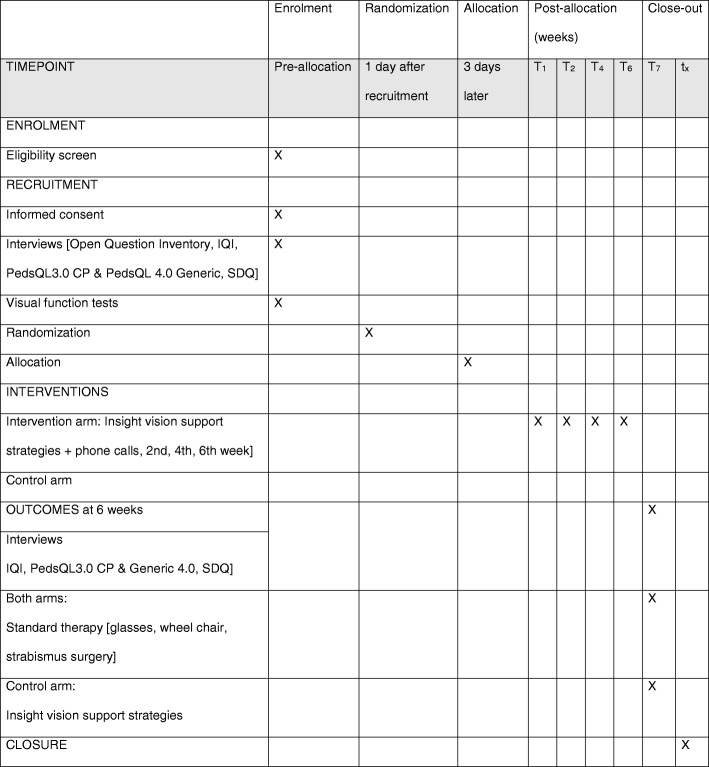
SPIRIT flow diagram. CP cerebral palsy, IQI Insight Questions Inventory, PedsQL Paediatric Quality of Life Inventory, SDQ Strengths and Difficulties Questionnaire

This is a parallel group, double-blind clinical trial with equal arm allocation, with a superiority design. This prospective community study will be conducted in 18 local government areas in Cross River State, Nigeria. The target population are children with CP, identified in the community by key informants and confirmed by a paediatric neurologist (see below). Figure 1 shows the SPIRIT flow diagram for the effectiveness of visual support strategies for visual impairment in children with CP showing the enrolment, intervention and assessment.

### Eligibility criteria

The inclusion criterion is children aged 4 to < 16 years diagnosed with CP (by a paediatric neurologist using standardized diagnostic criteria) [5] of any type or severity and their carers.

Exclusion criteria are children with other causes of motor disorders, children whose carers refuse to participate, and children with CP who have debilitating illness and require immediate medical care.

### Intervention

#### Intervention arm: selected IQI vision support strategies

The intervention is the application of some of the IQI visual support strategies (Additional file 1) chosen by the carers based on the “often” or “always” response to the 52 IQI questions generated by the Insight software. The social worker will explain and discuss the tailored strategies with the carer, who will be asked to select eight visual support strategies which they consider to be the most important, relevant and practical to implement. They will be asked to select one to three of the most important to start with, and those which could be implemented thereafter. They will be advised to practise the intervention three times a day. Carers will be given a list of all the strategies they have selected. To improve adherence and as part of the intervention, carers will be contacted by a telephone call every 2 weeks (at 2, 4 and 6 weeks). Each telephone interview will be conducted by the social worker to assess progress in implementing the strategies, to identify challenges in implementation, and to remind them of the additional strategies they selected and encourage their application. A checklist of questions will be used.

#### Control arm: no IQI vision support strategies

No intervention will be given during the 6 weeks after recruitment. After week 6 follow-up assessment has been completed, carers of children in the control arm will be offered vision support strategies based on their response to the IQI.

### Follow-up

The trial period is 6 weeks. During the trial, telephone calls will be made at weeks 2, 4 and 6.

### Outcome measures

The primary outcome is a comparison of the change in PedsQL 4.0 generic and 3.0 CP mean scores between baseline and follow-up at 6 weeks between the two arms of the trial. The PedsQL is being used as the primary outcome measure because there is a clear conceptualization of the health-related quality of life construct for children in general and those with CP, which reflects concepts in the assessment of PVD.

Secondary outcomes include change in functional vision, using the mean scores in the IQI and the SDQ between baseline and 6 weeks between the two arms of the trial.

### Sample size calculation

Data from a pilot study in Bangladesh were used in the sample size calculation. In the pilot study of 180 children with CP, an improvement in quality of life as measured by PedsQL 4.0 generic of approximately 0.3 standard deviations (SD) was seen in the study population [21]. Using the Altman nomogram [36], a sample size of approximately 370 children with CP, with 185 children in each arm, is needed to detect an effect size of 0.3 SD with 80% power and at 95% confidence, allowing for 5% loss to follow-up.

### Identification of study population and recruitment

Recruitment will take place over 12 months between 2017 and 2018. In the first stage, children with CP will be identified in the community using the key informant method. In the second stage, eligible children will be recruited into the trial. Table 1 gives a general overview of the research stages.

**Table 1:** Overview of methods at different stages in the trial

	Before randomization	After randomization	
		Intervention arm	Control arm
Before recruitment			
Identification of children with motor impairment in the community	X		
Examination to confirm presence of CP	X		
Examination to exclude those ineligible	X		
Eligibility confirmed	X		
Recruitment with informed consent	X		
After recruitment			
Ocular examination (visual function)	X		
Distance visual acuity (Lea symbols)	X		
Near vision acuity (Lea symbols)	X		
Visual acuity (Peek/Peekaboo)	X		
Grating acuity (Lea paddles)	X		
Contrast sensitivity (Hiding Heidi)	X		
Colour vision assessment (Ishihara chart)	X		
Visual fields assessment (Lea Flicker wand 280,000)	X		
Stereopsis (Butterfly stereo acuity test)	X		
Extraocular motility assessment	X		
Vergence test	X		
Smooth pursuit assessment	X		
Saccades assessment	X		
Ocular alignment	X		
Assessment of accommodation (near pupillary reaction)	X		
Cycloplegic refraction	X		
CVI/PVD assessment (functional vision assessment)	X		
Visual fixation	X		
Visual guidance, colour, visual memory	X		
Visual guidance, 3D recognition of concrete objects	X		
Visual recognition and line orientation	X		
Visual recognition of size, length and direction of lines	X		
Visual attention	X		
Visual search	X		
Facial recognition	X		
Baseline interviews	X		
Open-questions inventory	X		
IQI inventory (closed questions)	X		
PedsQL 3.0 CP	X		
PedsQL 4.0 CP	X		
Strength and Difficulties Questionnaire	X		
Data management	X		
Data entered into database	X		
Randomization	X		
At baseline			
IQI inventory		X	X
Strategies selected by carers		X	–
Telephone calls at 2, 4 and 6 weeks		X	–
At 6 weeks			
PedsQL 3.0 CP		X	X
PedsQL 4.0 generic		X	X
Strength and Difficulties Questionnaire		X	X
IQI Inventory (closed-questions inventory)		X	X
Strategies selected by carers		–	X

*CP* cerebral palsy, *CVI* cerebral visual impairment, *IQI* Insight Questions Inventory, *PedsQL* Paediatric Quality of Life Inventory, *PVD* perceptual visual disorders

#### Identification of children with CP

A community-based sample of children with CP will be identified and recruited to reduce the selection bias inherent in health facility-based studies. The key informant methodology (KIM) has been shown to be effective for identifying children with severe visual impairment and blindness and children with disabilities [37, 38], including those with CP, in lower- to middle-income countries. The ten-question questionnaire has been validated in an African community for use in the identification of children with neurodevelopmental disorders including CP [39]. The KIM involves identifying recommended, respected community members who have had no previous medical training (for example, community leaders, teachers, and local health or development workers). The key informants will undergo 1 day of training by the Principal Investigator (PI) using the ten-question questionnaire as a screening tool [39]. After training, they will use a range of approaches to identify and list children who might have CP over a 2-week period. Children they identify and their carers will be invited to a pre-agreed site, usually a primary health centre, for comprehensive history and detailed examination, including neurological examination and confirmation of the diagnosis and classification of CP by a paediatric neurologist using diagnostic criteria [5]. Children with CP who have debilitating illness will be referred immediately to the tertiary health facility at the University of Calabar Teaching Hospital and will not be recruited. The physiotherapist will assess each child’s functions using the GMFCS, MACS and CFCS, and determine the mobility aids required [10]. Children who need wheelchairs and ophthalmic interventions such as corrective glasses and strabismus surgery will be scheduled for intervention after the period of the study (it would not be feasible to provide these sooner than 6 weeks even if the children were not enrolled in the study).

Data on all eligible children (name, age and gender of the child, and address and phone number(s) of carers) will be entered into a password-protected database. Each child will be allocated a unique identification number.

#### Informed consent and assent

Written informed assent will be obtained from carers and consent from children who are old enough and who are able to understand what is being asked of them. Consent to participate in the interviews will also be taken from carers. Consent will be obtained after a full verbal explanation of the study has been given by a dedicated research officer and the key informant who identified the child, an information leaflet has been offered, and time allowed for questions. The right of the child or carer to refuse to participate without giving reasons will be respected, and it will be made clear that carers and/or their child are free to withdraw from the trial at any time without giving reasons with no repercussions for them or their child. If carers withdraw before randomization, another child will be recruited. If a carer withdraws their child during the study, reasons will be sought. Before giving assent, carers will be informed that, should they subsequently withdraw, all the data collected prior to their withdrawal will be maintained and used for the purposes of the study. The PI will be responsible for ensuring that all participants are protected and can participate voluntarily in an environment free from coercion or undue influence.

### Visual function tests

The CVI/PVD research questionnaire will be completed by the ophthalmic nurse, optometrist and the paediatric ophthalmologist. Sub-sections of the form include demography, history, visual and motility assessments, and ophthalmological examinations including anterior segment examination will be performed with a portable slit lamp (Reichert Technology) and dilated posterior segment examination will be performed by an ophthalmologist using a binocular indirect ophthalmoscope (Appassamy) with a 28D or 20D lens. Fundus photographs will be taken using a Horous fundus camera. Visual fields will be assessed using the Lea wand. The following criteria for prescribing glasses will be used: myopia ≥ −2.0D, hypermetropia ≥ +4 D, and astigmatic cylinder ≥ −2.0D. Table 2 shows the methods of assessment of visual function.

**Table 2:** Visual function and tests to be performed

Visual function	Test	Performed by
Presenting distance visual acuity	Lea symbols	Optometrist
Presenting distance visual acuity	Peek visual acuity test; Peekaboo visual acuity test	Optometrist
Presenting near visual acuity Reading	Lea symbols Goodlite children’s reading chart	Optometrist Optometrist
Grating acuity	Lea paddles	Optometrist
Contrast sensitivity	Hiding Heidi	Optometrist
Colour vision	Acquired and congenital colour chart	Optometrist
Visual fields	Lea Flicker Wand 280,000	PI
Stereopsis	Butterfly stereo acuity test with Lea symbols	Optometrist
Eye movements		PI
Vergence	Observation of binocular movements of the eye, i.e. convergence and divergence, using a fixation target	PI
Motion processing: smooth pursuit (horizontal and vertical)	Uncooperative or inattentive children are tested by slowly rotating a mirror that measures 10 cm by 15 cm held before their eyes Verbal children: the patient is asked to track the tip of a blue ball point pen held 1 m before the eyes with the head still; the target is moved at a low, uniform speed	PI
Motion processing: saccades	Non-verbal: the infant or child is enticed to look at the tester’s face; when the fixation is in the midline, one of the objects is presented at about 20–30 cm from the midline and the infant or child is again enticed to look at the tester’s face after which the other object is presented on the other side. Verbal: the patient is asked to look at the examiner’s nose and then at the examiner’s finger to the left or right of central fixation only upon verbal command.	PI
Strabismus	Corneal reflex test and modified Krimsky test	PI
Accommodation	Near pupillary reaction method	PI
Cycloplegic refraction	Cyclopentolate 2% + phenylephrine 2.5% Zeiss autorefractor/keratometer Manual objective retinoscopy	Optometrist PI

*PI* Principal Investigator

### Assessment of CVI/PVD

CVI/PVD examination will be conducted using several tests [40–43]. Table 3 describes the tests used to assess CVI/PVD. Each observation has a score based on the ability or inability of a child to perform, and all will be undertaken by the researcher (RD). The modified IQI, a 52-item symptoms-based inventory, will be administered to each carer to assess the nature of visual difficulties including CVI/PVD in the children. There are seven sections, and responses to each question will use a five-point Likert scale to describe whether a child has problems (“never”, “rarely”, “sometimes”, “often”, or “always”, including “not applicable”). These responses will be awarded numbers from 1 to 5, respectively. The questions in each section are designed to identify CVI/PVD. Visual tasks involve both the dorsal and ventral visual streams functioning together; however, the activities asked about in sections 1, 2, 3, 4 and 5 are designed to be illustrative of mainly dorsal visual stream abilities whilst sections 6 and 7 rely more on ventral stream function. The number of subjects with an arbitrarily set mean score of 3 or more for each or any section will be recorded, indicating on average that the child had problems with the tasks being asked about “sometimes”, “often” or “always” and therefore had PVD. Questions with more than half of respondents reporting “not applicable” will be excluded.

**Table 3:** Assessment for CVI/PVD, corresponding test, observations, and grading by Principal Investigator

Subcategory of CVI/PVD	Test	Observation	Grading
Visual fixation	A fixation light is presented before both eyes at a distance of 0.75 m	Central, sustained or maintained	Present (central, sustained and maintained) or absent
Visual guidance, colour, eye–hand coordination and visual memory	Lea 3D puzzle, coloured	Eye–hand coordination and visual guidance of movement: watching the movement a child makes with their hand, elbow or forearm when asked to turn over a piece of puzzle; the ability to place the puzzle pieces correctly The capacity to orientate the shape is subjectively viewed Short-term memory for localization: can a child place a piece of puzzle in the correct place after being distracted	Easy, difficult, cannot do and why, or not applicable
Visual guidance and 3D recognition of concrete objects	Lea 3D puzzle, black and white	Eye–hand coordination and visual guidance of movement: watching the movement a child makes with their hand, elbow or forearm when asked to turn over a piece of puzzle 3D assessment of an object; ability to place the puzzle pieces correctly Short-term memory for localization: can a child place a piece of puzzle in the correct place after being distracted	Easy, difficult, cannot do and why, or not applicable
Visual recognition and line orientation in three dimensions (vertical, horizontal, oblique) and eye–hand coordination	Lea mailbox game	Visual perception of line orientation done with the fingers, hand, or forearm: • the child is asked to drop a card through the slot of the LEA Mailbox Game • the child is asked to match the orientation of the slot	Easy, difficult, cannot do and why, or not applicable
Visual recognition of differences in size, length and direction of lines	Lea rectangles game	Size, length perception: • watch the orientation of the hand and fingers as the child grasps a block • can the child place a block of the same length on top of another? • can the child appreciate short and long as a change in length? • response to the enquiry concerning whether a correct or an incorrect arrangement looks the same	Easy, difficult, cannot do and why, or not applicable Yes, no, not sure, do not know, not applicable
Visual attention	Identification of self-reflection in the mirror test Pick-up test: picks up a small ball of uniform size (1 mm) placed on a patterned surface	Identification of self-image The distance at which the child loses interest in self-image Searches for and identifies the ball	Alert and attentive, alert but not attentive, not alert, not attentive Documentation of the distance at which attention is lost; easy, difficult, cannot do and why, or not applicable
Visual search	Pick-up test: picks up a small ball of uniform size (1 mm) placed on a plain or patterned surface	Searches for and identifies the ball	Easy, difficult, cannot do and why, or not applicable
Facial recognition test	Heide expression test	Interpretation of facial expressions: can the child interpret the facial expression? Can the child identify a similar facial expression?	Yes, no, cannot do, why or not applicable Easy, difficult, cannot do and why, or not applicable

*CVI* cerebral visual impairment, *PVD* perceptual visual disorders

After examination, participants and their carers will disperse to their homes and await further contact by telephone call.

### Randomization

Baseline examinations are performed before randomization so that stratification and blocking can occur and also so that those collecting the baseline data are masked to the allocation.

#### Sequence generation

The database of children recruited is accessible to the researcher and contains information on their unique ID, age, sex and GMFCS score. The database will be sent to the data analyst in the University of Calabar Teaching Community Medicine Department at the end of the examination per consecutive local government area. Children will be stratified and blocked by age groups 4–9 and 10 to < 16 years and by GMFCS levels 1–3 and 4–5. The randomization sequence will then be generated by the data analyst using the Stata 11 programming syntax for block randomization of patients into the treatment and control group. The randomization database will be password protected, and only accessible to the data analyst. The randomization database holds data on the patient’s unique ID, block number, block sizes and treatment versus control groups. The data analyst will merge the randomization database with the patient database using the patients’ unique ID. The result of randomization in the combined dataset will be sent to the research officer for the purposes of patient enrolment/allocation into the treatment arm of the randomized clinical trial.

#### Allocation concealment and implementation

Carers of children assigned to the treatment arm of the trial will be contacted by an independent research clerk, who is masked to the baseline examination, by telephone call and also by the key informant within 3 days of recruitment. Carers will be invited to visit the same primary health centre where the baseline examination was conducted at an agreed time convenient to the carers. At this visit, the intervention will be selected by the carers and explained to them by the social workers.

### Contamination and masking

It is unlikely that contamination will occur as allocation is not being done on the same day as the examination, and carers of children in intervention and control arms are unlikely to meet after the baseline assessment. The research assistant who is not part of the initial examination will place telephone calls to carers within 3 days of the examination. The data analyst involved in the sequence generation is not involved in any of the field work. No member of the team collecting baseline data will be involved in explaining the intervention nor in collecting outcome data. Parents or carers who do not respond to telephone calls will be personally contacted by the key informant. Allocation is concealed to the PI and all the members of the examination team. The randomization sequence and allocation is computer generated and cannot be changed.

The week 6 outcome interviews will be performed by a different set of researchers from those who collected pre-intervention data. This follow-up assessment will be from a mix of children in the intervention arm and in the control arm attending on the same day.

### Data collection methods

Interviewers will be trained for 6 weeks on data collection using the following study instruments: PedsQL3.0 CP, PedsQL4.0 generic, SDQ, IQI and strategies, and strategy follow-up form.

Data will be collected at baseline and at the end of follow-up after 6 weeks. Outcome data will be entered into one Microsoft Excel database by a masked medical records officer who has no knowledge of, or access to, identifiable participant information or treatment assignment.

#### Open ended and IQI

All carers of children with CP will be interviewed with both the open-ended questions inventories and then the modified IQI at baseline, and at the end of 6 weeks only with the modified IQI. Details of the IQI are shown above (‘Assessment of CVI/PVD’).

#### Quality of life methodology

For the PedsQL 4.0 generic tool and the PedsQL CP 3.0 module, the parent’s proxy form will be used.

The 23-item PedsQL 4.0 generic core scales encompass: 1) physical functioning (eight items); 2) emotional functioning (five items); 3) social functioning (five items); and 4) school functioning (five items). The 35-item PedsQL 3.0 CP module is composed of seven scales: 1) daily activities (nine items); 2) school activities (four items); 3) movement and balance (five items); 4) pain and hurt (four items); 5) fatigue (four items); 6) eating activities (five items); and 7) speech and communication (four items). The parents will be given a five-point Likert response scale (1 = “never”, 2 = “almost never”, 3 = “sometimes”, 4 = “often”, 5 = “almost always”) to choose one single option. The results will be entered on the PedsQL form. The results from the questionnaire will be transferred to an Excel sheet to number 0 to 4, respectively. To interpret the scores, items are reverse scored and linearly transformed to a 0–100 scale, so that higher scores indicate better health-related quality of life. To reverse the score, the 0–4 scale items will be transformed to 0–100 as follows: 0 = 100, 1 = 75, 2 = 50, 3 = 25, 4 = 0. To create the scale scores, the mean will be computed as the sum of the items over the number of items answered (this accounts for missing data). The number of missing values in the scale will be summed and the item scores divided by the number of items in the scale minus those that were missed. The mean psychosocial health summary score will be computed as the sum of the items over the number of items answered in the emotional, social, and school functioning scales. The total scale score, for both the generic and CP modules, will be obtained by computing the mean as the sum of all the items over the number of items answered on all the scales [44]. If more than 50% of the items in the scale are missing, the scale scores will not be computed. If 50% or more items are completed then they will be used to impute the mean of the completed items in a scale according to PedsQL guidelines.

#### Strength and Difficulties Questionnaire

The SDQ is a brief emotional and behavioural screening questionnaire for children and young people; the interview will be conducted after the PedsQL 4.0 generic and 3.0 CP module and administered at the examination site. The SDQ has 25 items on psychological attributes that are divided between five scales: emotional symptoms, conduct problems, hyperactivity/inattention, peer relationship, and pro-social behaviour. The parents/carers will be given instructions to give their answers on the basis of the child’s behaviour over the last 6 months. The reports will be computer generated and optimized by the ‘youthinmind’ team.

### Data management

The Data Monitoring Committee, which is independent of the sponsors, will provide oversight of the data collection process and will meet after recruitment has been completed in each local government area. Two optometrists and two ophthalmologists will undergo competency training in clinical examinations to ensure standardization of data collection methods and in completing the ophthalmic examination questionnaire. Two nurses will be trained on history taking using the open questions inventory, and questionnaires including the PedsQL 4.0 generic and 3.0 CP module and the SDQ. Four social workers will be trained to administer the IQI and strategies and follow-up form. Two nurses will be trained to be part of the follow-up team for the week 6 follow-up assessment. Successful completion of competency training for all research staff will be confirmed by a competency checklist.

Participant data will be stored securely, and their confidentiality protected. Each participant will be given a unique trial number at the end of the examination and this will be used throughout the study to identify all data relating to the participant. Data collected by all members of the research team will be entered on paper then transferred to a Microsoft Excel data sheet on a protection compliant drive in the research office. Questionnaires and inventory questions will be assessed at the end of the research day. Where there are missing data, every attempt will be made to complete the data. Data will be entered the following morning. Data entry errors will be corrected at the end of each following day. Data will be updated to the Chief Investigator every day that data are collected. All patient-identifiable information will be kept on encrypted computers in a secure office, with access limited to authorized members of the research team. All data will be archived in secure archive facilities in the London School of Hygiene & Tropical Medicine (LSHTM) for 10 years. After this time, all documentation will be kept in a general data pool in LSHTM. The final dataset will initially be available only to the study team. After the results of the trials have been reported, the trial data will be made publicly available through the LSHTM repository.

### Statistical methods

Missing data will be addressed at the analysis stage. Statistical analyses will be conducted with STATA (version 14.0) as an intention-to-treat analysis. Our descriptive statistics will include means and SD, medians and interquartile ranges for continuous variables, and the number and proportions for categorical variables as appropriate.

We will compare the two intervention groups at baseline regarding characteristics and demographics using two-tailed Student’s *t* tests and the effect size to test the strength of the difference. Variables that differ between the groups at baseline will be considered as possible confounders and adjusted for in subsequent analyses. The alpha level will be set at 0.05 for all comparisons.

We will use the unpaired (two-sample) *t* test for comparing the change in PedsQL 4.0 and 3.0 CP module score and IQI score in arm 1 versus arm 2. Skewed data will be analysed with non-parametric test alternatives (e.g. we will use the Mann-Whitney *U* test instead of Student’s *t* tests if data are skewed). We will use the paired *t* test for comparing mean scores of PedsQL CP 4.0 and 3.0 CP module scores before and after intervention. We will use the paired *t* test for comparing mean IQI scores before and after the intervention. Potential effectors of PedsQL 4.0 and 3.0 CP module and IQI (visual function) outcome, such as socio-demographics and all medical and clinical variables (GMFCS, MACS, CFCS, grade of vision, key CVI/PVD examinations and types of CP) will all be investigated pairwise and those found to be correlated will be entered into a multivariate regression model. We will undertake subgroup analysis on the type of CP and what level of GMFCS showed improved quality of life, and analysis by socio-demographic factors, age, sex, type of CP, GMFCS, MACS, CFCS, visual acuity, type of PVD by insight domain, and association with ocular examinations.

### Monitoring

#### Adverse events

Reported adverse events such as pain or distress will be carefully monitored throughout the trial, although minimal risk is anticipated. Contacts for reports of adverse reactions will be put in place and carers advised to report to the study centre at the Calabar Childrens’ Eye Center. Reporting of adverse events will follow LSHTM guidelines on recording, managing and reporting adverse events for behavioural interventions [45].

#### Loss to follow-up

Participants who do not respond to the 2-weekly telephone calls by week 6 will receive a home visit from the key informant, and the end of trial questionnaires will be administered at their home by a member of the research team. Participants who are lost to follow-up will be included in the analysis.

#### Audit

Data will be reviewed at the end of the field work on a daily basis. Random comparisons between entered data and filled protocols will be performed after each five entries. This will be done independently of the sponsors. The study may be subject to audit by the LSHTM under their remit as sponsor, the Study Coordination Centre and other regulatory bodies to ensure adherence to Good Clinical Practice (GCP).

## Discussion

The Effectiveness of Visual Support Strategies for Visual Impairment in Cerebral Palsy (EVSSCP) study will be the first randomized controlled trial to investigate the effect of tailored visual support strategies for the treatment of PVD in children with CP. Evidence-based management strategies for children with this problem are urgently needed.

Considering the lack of awareness of visual support strategies in the treatment of PVD and the need for knowledge dissemination in this area, this trial should lead to a significant advance in the management of this condition.

All the children with CP are being recruited to the trial, not just those with confirmed CVI. The reason for this is that subtle visual problems are common in CP and since detailed visual assessment is not always possible in practice it would be useful to have a tool that can be given to all CP families. The tool is self-titrating in that if no visual problems are reported in the IQI then no strategies will be given. This could potentially reduce the power of the trial, but if the tool is found to be useful for all children with CP then it will be clinically more useful.

Although pilot data from Bangladesh has been encouraging, there has been no randomized controlled trial that measures the effects of potentially effective visual support strategies in habilitating children with PVD. If it were shown to be effective then it could be scaled up as a community-based intervention.

### Limitations

The EVSSCP trial is planned to be carried out as a single country study and can, therefore, only be seen as a preliminary study in Africa. In this study there are limitations in the use of some examination methods [46].

### Trial status

This is protocol version number 2 of 18/09/2016, and recruitment began on 4 December 2017. Recruitment is expected to be completed by the end of July 2018.

## Additional file

Additional file 1: Modified Insight Questions Inventory (IQI) and tailored visual support strategies. (DOCX 56 kb)

## Data Availability

The data that will be generated will be available from the LSHTM repository, but restrictions apply to the availability of these data, and so are not publicly available. Data are, however, available from the authors upon reasonable request and with permission of LSHTM.

## Abbreviations

CFCS: Communication Function Classification Scales
CP: Cerebral palsy
CVI: Cerebral visual impairment
EVSSCP: Effectiveness of Visual Support Strategies for Visual Impairment in Cerebral Palsy
GMFCS: Gross Motor Function Classification System
IQI: Insight Questions Inventory
KIM: Key informant methodology
LSHTM: London School of Hygiene & Tropical Medicine
MACS: Manual Ability Classification System
PedsQL: Paediatric Quality of Life Inventory
PVD: Perceptual visual disorders
SDQ: Strengths and Difficulties Questionnaire
