# Supplementary material for: The effect of visual support strategies on the quality of life of children with cerebral palsy and cerebral visual impairment/perceptual visual dysfunction in Nigeria: study protocol for a randomized controlled trial
Source: Trials. 2019 Jul 10;20:417. doi: 10.1186/s13063-019-3527-9 (PMC6617659; doi:10.1186/s13063-019-3527-9)
Supplement: Supplementary file 1 — Modified Insight Questions Inventory (IQI) and tailored visual support strategies. (DOCX 56 kb) [file 13063_2019_3527_MOESM1_ESM.docx]

Additional file 1

Modified Insight Question Inventory and tailored visual support strategies

|  |  |  |
| --- | --- | --- |
| Modified Insight Questions Inventory | |  |
| About your child | | |
| Child's forename: | | Surname: |
|  | |  |
| D.O.B.: | | Gender: |
|  | |  |
| Instructions to parents | | |
| These questions are designed for a range of ages, so some questions may seem odd. Your child may have difficulty with some behaviours listed below but not others – this is normal. | | |
| For each of the items listed, please could you click on the box next to it and select which best fits with your child’s present behaviour: never/ rarely/ sometimes/ often/ always/ not applicable (NA). | | |
| 1 | Does your child fall or stumble onto things on the floor? |  |
| 2 | Does your child find it hard to walk down the hill or stairs? |  |
| 3 | Does your child want to fall or does it seem as if your child does not notice when the foot path or pavements go up or down? |  |
| 4 | Does your child get frightened not knowing what to do at the top of a slide or hill or in front of a gutter? |  |
| 5 | Does your child look down when they cross floor boundaries, for example where tiles meets the cement floor? |  |
| 6 | Does your child leave food on their plate? If so, is this on the near or far side? |  |
| 7 | Does your child leave food on their plate? If so, is this on the left or right side? |  |
| 8 | Does your child have any problem stepping into the bath, apart from any problems with balance? |  |
| 9 | Does your child find it hard to find the beginning of a line or the next word when reading, or miss pictures or words on one side of a page (left /right)? |  |
| 10 | Does your child walk, move out in front of traffic (coming from left/ right / both)? |  |
| 11 | Does your child hit his/her body into door frames or partly open doors (left/right/both) when trying to pass through? |  |
| 12 | Does your child have difficulty seeing things from a moving vehicle? |  |
| 13 | Does your child find it hard to identify things (or know what has just passed by) that are moving pass quickly, such as children, small animals? |  |
| 14 | Does your child find it hard to see moving water i.e. to know when the cup is full? |  |
| 15 | Does your child find it hard to follow a cursor on the computer screen? |  |
| 16 | Does your child avoid watching fast moving video CD, and prefer to watch slow moving video CD programmes? |  |
| 17 | Does your child find it hard to catch a ball? |  |
| 18 | When walking, does your child hold onto your clothes, dragging down? |  |
| 19 | Does your child find uneven ground hard to walk over? |  |
| 20 | Does your child bump into low furniture, for example a small stool? |  |
| 21 | Does your child get angry if furniture is moved? |  |
| 22 | Does your child explore floor boundaries (for example, where tiles meets a cement ) with their foot before crossing |  |
| 23 | Does your child reach incorrectly for objects (i.e. reaches beyond or around the object or when picking it up, grasp |  |
| 24 | Does your child find it hard to see something pointed out in the distance? |  |
| 25 | Does your child find it hard to recognize a close friend or relative standing in a group? |  |
| 26 | Does your child find it confusing or difficult when playing team games with a lot of players, epecially if there are a lot of players and everyone is moving? |  |
| 27 | Does your child have difficulty finding an item on the kitchen or cupboard shelf e.g. finding the salt or pepper? |  |
| 28 | Does your child find it hard to spot an item of clothing in a pile of clothes? |  |
| 29 | Does your child find it hard to find something they want in a full toy cartoon, shoe in a box of shoes etc? |  |
| 30 | Does your child find it hard to find anything when the object is in a place with the same color of the place? E.g. a white tee shirt on a white bedsheet? |  |
| 31 | Does your child get lost in places where there is a lot to see, e.g. in a crowded market or shopping centre? |  |
| 32 | Does your child find copying words or drawings time consuming and difficult? |  |
| 33 | Does your child have difficulty reading crowded text on paper or on a computer screen, but can cope better if some of the text is covered or taken away? |  |
| 34 | Does your child find it hard to notice or look for letters on a keyboard but knows the alphabet? |  |
| 35 | Does your child sit closer to the television than about 30 cm? |  |
| 36 | Does your child find it difficult to concentrate or focus for more than 5 minutes, or after being distracted do they find it difficult to get back to what they were doing? |  |
| 37 | Does your child react angrily when other children cause distractions? |  |
| 38 | Does your child bump into things when they are walking and talking at the same time? |  |
| 39 | Does your child miss objects that are obvious to you because they are different from their background and seem to ‘jump out’ e.g. a bright ball in the grass? |  |
| 40 | Does your child get upset in busy places, such as markets or shopping centres? |  |
| 41 | Does your child find it difficult to recognise close relatives in real life? |  |
| 42 | Does your child find it hard to recognise close relatives from photographs? |  |
| 43 | Does you child mistakenly identify strangers as people known to them? |  |
| 44 | Does your child have difficulty understanding the meaning on your face? |  |
| 45 | Does your child find it hard to name common colours? |  |
| 46 | Does your child have difficulty naming basic shapes such as squares, triangles and circles? |  |
| 47 | Does your child find it hard to recognise well known objects such as the family house or the church building? |  |
| 48 | Does your child find it hard to find their way around a well known environment, for example church? |  |
| 49 | Does your child find it hard to recognise an object if it is partially hidden or viewed from an unusual angle (such as a shoe under the bed with only the toe showing)? |  |
| 50 | Does your child find it hard to identify which shoe is right and which shoe is left? |  |
| 51 | Does your child find it hard to recognise people, or words, or objects if they are changed in any way, for example font of text, hairstyle? |  |
| 52 | Does your child find it difficult to identify where sound is coming from, for example if they shout for you in the house do you have to clearly tell them which room you are in, as they are unable to work out where you are from your voice alone? |  |

Modified Visual support strategies tailored to IQI questions

Q1 Trips / moves over toys and obstacles on the floor:

❶ Keep floor space tidy and clear of things. ❷ Make a secret code to use when you are out and about. A tap on your child's shoulder could mean "Watch out! There is danger in the room." ❸ Encourage your child to use a stick (plucked from a tree) to feel the height of the ground ahead. The stick can also be used to identify and guide him around blockades. ❹ Try other ways to help your child walk over uneven ground and around Blockades. For example, offer your child your arm to hold. Suggest he holds onto the belt or clothing of someone who is walking with him, or encourage him to hold onto a wall. ❺ Tell your child about things blocking his path and dangers he could miss due to his reduced field of view. (For example "there is a plate on the floor in front of you.") ❻ when your child comes to an object on his way teach him to slow down, look, look again and then go - "Slow, Look, look again, Go". This can be used inside and outside.

Q2 Has difficulty walking down stairs or slopes:

❶ Use extra supports so your child can feel the height and position of steps. For example hand rails at the correct height (on both sides where possible) or holding onto an adult. ❷ Tell your child to slow down and hold on to the hand rails when he comes across steps. ❸ Give your child an agreed warning when you come across steps or stairs, this could be a sound or touch. ❹ Use bright lighting or spot lights on stairs. This is most important at the top, the bottom and on the landing. This will create shadows and make the steps clearer. ❺ Try using a torch at night. ❻ Tell your child to look down at his feet when he goes down stairs. ❼ Plain floor covering (e.g. laminate or plain carpet/tiles) can make it easier to walk down stairs. Patterns cause distractions, and can act like obstacles. ❽ Try to keep walls plain. For example use plain not patterned curtain and don't have too many pictures on the wall. ❾ Let your child go first or last when using stairs. This is more important if there is a crowd of people (such as at school or in a shopping centre). ❿ Encourage your child to receive assistance. ⓫ Let your child have lots of time to practice. ⓬ Arrange for your child to leave class a little earlier or later than his class mates This will allow him to miss the rush and crowds of other people on the stairs. ⓭ Talk to your child's school about having his classes on the ground floor. ⓮ Highlight the edge of each step with a bright colour. Or put a dot in the middle of each step to show where his foot should go. ⓯ Use a colour to mark the area beyond the top and bottom of the stairs. This may help to show more clearly where the stairs start and finish. ⓰ Give your child extra support when he is going up or down stairs/ steps.

Q3 Trips or are unaware of the edges of pavements going up/ down:

❶ Give your child reminders. For example "There is a pavement to go up in 3 metres." ❷Use Pedestrian Crossings where available because pavement stones are lower at these points. ❸ Give your child reminders to look down.

Q4 Appears to 'get stuck' at the top of a slide or hill:

❶ Encourage your child to practice around the house trying to cross small gutters and playing on small play slides and/or by lying on his tummy on a scooter board or skate board. Some children choose to go down slides head first. Do not stop this, but make sure it is safe. Children may do this because the upper part of their field of view is being used in this situation. ❷ Give additional verbal information.

Q5 Looks down when crossing floor boundaries e.g. where lino meets carpet or tiles:

❶ Give your child extra hints and ideas. For example, "The floor changes here." ❷ Try not to use patterned carpets or tiles, plain carpets and floor surfaces may help make changes easier to see. ❸ Make sure floors are well lit where one surface stops and another starts. For example, wood to carpet/tiles at a doorway. ❹ Try using coloured tape to mark a change in surface, this can make it easier for your child to spot the changes. For example, where carpet/tiles changes to tiles. ❺ Suggest your child pushes a push toy ahead of him while walking.

Q6 Leaves food on their plate (near, far):

❶ Put your child's favourite foods on the part of his plate that is usually forgotten. ❷ At meal times make sure your child turns his plate. This could be done when you tell him, ❸ Put your child's plate on a spinning base, like those found in a Microwave. ❹ Put food on plain, clear or transparent plates or separate plates. ❺ Try to avoid using gravy or sauces, as this can make the different foods on a plate look like one. ❻ Sit your child on a lower seat at meal times. This may give a better view of whole plate. ❼ If you put more than one type of food on a plate make sure each food is a different colour or in a different plate to the others. This may help your child to spot the different foods.

Q7 Leaves food on their plate (left, right):

❶ Put your child's favourite foods on the part of his plate that is usually forgotten. ❷ At meal times make sure your child turns his plate.. ❸ Put your child's plate on a spinning base, like those found in a Microwave. ❹ Put food on plain, clear or transparent plates or separate plates. ❺ Use different types and colours of food. This may make each food easier to spot. ❻Try to avoid using gravy or sauces, as this can make the different foods on a plate look like one. ❼ Sit your child on a lower seat at meal times. This may give a better view of whole plate. ❽ If you put more than one type of food on a plate make sure each food is a different colour to the others. This may help your child to spot the different foods.

Q8 Has difficulty stepping into the bath, which is not related to balance:

❶ Put one bathmat outside the bath and one inside. They should be bright, and a different colour to the bath. ❷ Put stickers/notices on the edge of the bath to make it stand out.

Q9 Has difficulty finding the beginning of a line or the next word when reading, or misses pictures or words on one side of a page (left/right):

❶ Ask your child to point to each word with his finger. ❷ View only one line of a book at a time. This can be done using a piece of card with a viewing window cut out of it. This reduces the amount of text on view and can be moved from line to line. ❸ Make the font size bigger and/or use double spacing between lines. ❹ Make sure only a small amount of information is shown to your child at a time. For example, a small amount of text on a page, a small amount of information on the board, enlarge the size of the print. ❺ Make the print in text books bigger by using a photocopier to enlarge each page. ❻ Block out extra bits of work or pictures on a page. This can be done using a blank piece of card or the back of your hand. ❼ Use a magnifying glass. ❽ Find the font size and number of words per page, and line, that your child prefers to work with (not the smallest he can read). Make sure work is given to your child in this size and layout. ❾ Make a special work book by scanning photos, words and subjects that your child likes. ❿ Mark the point where text starts so the eyes are drawn to it. This can be done with a ruler, the back of your hand or a brightly coloured dot. ⓫ Use a raised book stand for reading, for example a recipe book holder. ⓬ Practice reading using items that your child enjoys. For example, The children's newspaper "First news". ⓭ See if the class reading book is available as a large print book. ⓮ Break text into small bits. This can be done by enlarging text on a photocopier and then cutting it into small sections. ⓯ Use a computer programme which shows one word at a time.

Q10 Walks out in front of traffic:

❶ Give your child extra hints. For example "We must wait here while we check it is safe to cross." ❷ When teaching your child to cross the road, teach him to cross at pedestrian crossings. ❸ Remind your child to listen carefully for cars, as well as looking, when he is crossing the road. ❹ Teach your child to turn his whole body from side to side when he looks for cars. You can encourage him by doing the same thing. ❺ Give additional supervision and guidance at roads.

Q11 Bumps into doorframes or partly open doors (left/right/both):

❶ Give extra hints. For example, "There is a door coming up in 3 metres." ❷ Help door frames stand out by painting doors frames and skirtings a bright or contrasting colour from walls. ❸ Replace doors with a beaded curtain. ❹ If your child always bumps into the same side of the door frame put a bright mark or picture on that side of door frame. Place the mark or picture at your child's eye level.

Q12 Has difficulty seeing scenery from a moving vehicle:

❶ Give extra reminders. For example, "There is a church coming up on your left." ❷ If your child doesn't like travelling in the car try asking him to wear wrap around sunglasses. This may reduce the feeling of movement and visual input. ❸ When you are out and about video interesting things on a camcorder or mobile phone. These can be discussed with your child later. ❹ Let your child sit in the front seat of the car.

Q13 Has difficulty seeing things which are moving quickly, such as small animals, children:

❶ Tell your child things that he might want to know. For example, "Your friend Jenny is at the gate in a pink jacket." ❷ Make sure friends and teachers tell your child who they are, especially if they meet in a busy area with lots of movement. ❸ Encourage your child to listen carefully to the voices of friends and family. Your child can then use a person's voice to help work out who they are. ❹ Encourage your child to shout for the person he wants to find. ❺ Encourage your child to use a mobile phone to call or text the person he wants to find. ❻ If your child is planning to meet up with friends or family make sure a meeting point has been arranged before hand. ❼ Teach your child to follow moving objects by moving his head as well as his eyes.

Q14 Has difficulty seeing moving water i.e. tends to over or underfill a cup:

❶ Ask your child to half fill cups. ❷ Use a clear plastic cup. ❸ Encourage your child to use a smaller cup that he can look into. ❹ Practice pouring the liquid together. Count how many seconds of pouring it takes to half fill the cup. Then use this as a guide. ❺ ❻ When pouring cool liquids teach your child put the thumb of his non-dominant hand pointing down into cup. Your child will feel the liquid on his thumb and know that the cup is nearly full. ❼ Teach your child to listen to the sound that is made when he fills a cup or glass. As a cup fills the sound of it filling changes. It becomes higher in pitch. ❽ Mark the cup with nail polish at the level it should be filled to.

Q15 Has difficulty following a cursor on the computer screen:

❶ Make the arrow head on the computer screen bigger. ❷ Switch the arrow head to a moving, instead of still, picture. This may make it easier for your child to find. ❸ Vary size of the computer/TV screen to suit your child's ability to see the whole picture, a larger screen may help if the child is needs a larger image, a smaller screen may help if the child finds it difficult to look at the whole picture at the one time.

Q16 Avoids watching fast moving TV, prefers to watch slow moving TV:

❶ Try a flat screen TV as your child may find this easier to see. ❷ Encourage your child to sit close to the TV. This may cut out some visual distractions. Some families have chosen to have an extra TV that their child can sit close to. ❸ Reduce the number of objects around the TV. i.e. photos, pictures, patterned wall coverings. These all act as visual distractions. ❹ Make sure the class teacher knows that many curriculum TV programmes can be difficult for your child to follow. This is due to the speed and content of the programme. Your child may be better off using the time for another task. ❺.

Q17 Has difficulty catching a ball:

❶ Practice catching skills with your child by throwing a balloon to each other. The balloon will move slower than a ball and may be easier for your child to catch. ❷ Put a little bit of rice / water in the balloon. The balloon will make a noise as it moves so your child can hear where it is. ❸ Use large, brightly coloured balls when playing catch or other ball games with your child. ❹ Use balls with sound or light effects when playing catch or other ball games with your child.

Q18 When walking, holds onto your clothes, tugging down:

❶ When walking together hold hands with your child. This works best if you hold your arm straight and slightly back. This gives your child a guide to the height of the ground ahead. ❷ Talk to your child as he moves, tell him what is coming up. For example "In three steps you will be moving onto carpet, "You need to lift your feet higher here as the ground is bumpy." ❸ Give your child extra physical support i.e. hand rails/banisters as required. ❹ Work on movement skills with activities and games. For example obstacle courses in the park, house or garden, riding, swimming or trampolining. ❺ Try other ways to help your child walk over uneven ground and around obstacles. For example, offer your child your arm to hold. Suggest he hold onto the belt or clothing of someone who is walking with him, or encourage him to hold onto a wall.

Q19 Finds uneven ground difficult to walk over:

❶ When walking together hold hands with your child. This works best if you hold your arm straight and slightly back. This gives your child a guide to the height of the ground ahead. ❷ Talk to your child as you walk together giving him reminders and instructions. For example, "You need to lift your feet higher here as the ground is rough." ❸ Encourage your child to use something to help with balance and tell him what is ahead. This could be a stone, bucket or walking pole. Make sure the aid suits your child's age and is something he has chosen to use. ❹ Make sure there is no change in height when one floor surface stops and the next begins. ❺ Encourage your child to wear white trainers or shoes to make his feet stand out. ❻ In play grounds/ play areas use coloured matting. Use different colours in different areas. ❼ Use coloured rubber flag stones to help different areas of ground in the playground or garden stand out more clearly. Make sure there is no height difference between each flag stone. ❽ Make sure there is safe ground cover in outside areas that are frequently visited. For example, floor games in the playground. ❾. ❿ Provide additional support and supervision if required.

Q20 Bumps into low furniture, such as a small table:

❶ Remind your child to change his head position and look down if he wants to look at an obstacle. ❷ Tell your child what is round about. For example "The table is just in front of you." ❸ If you are moving furniture around make sure your child is involved. ❹ Have less furniture in each room. This will make more space for your child to move around. ❺ Make sure the furniture is a different colour to the floor. Use plain floor and wall coverings. This will make the overall picture simpler for your child. ❻ Make sure you do not buy glass furniture or furniture with sharp edges.

Q21 Gets angry if furniture is moved:

❶ Remind your child to change his head position and look down if he wants to look at an obstacle. ❷ Tell your child what is round about. For example "The coffee/tea /dinning table is just in front of you." ❸ If you are moving furniture around make sure your child is involved. ❹ Have less furniture in each room. This will make more space for your child to move around. ❺ Make sure the furniture is a different colour to the floor. Use plain floor and wall coverings. This will make the overall picture simpler for your child.

Q22 Explores floor boundaries (e.g. lino/carpet) with their foot before crossing the boundary or finds floor boundaries difficult to cross:

❶ Tell your child about floor boundaries as he walks towards him. For example "The floor will change to tiles/carpet in 3 steps." ❷ Make sure there is good lighting in areas with floor boundaries (where one surface stops and the next starts). ❸ Use plain floor coverings. ❹ Give your child extra support if required. For example your arm, a rail, or the wall. ❺ Arrive early at new places. This may help your child get to know the new surroundings and find points where the floor surface changes.

Q23 Reaches incorrectly for objects, i.e. reaching beyond or around the object or when picking up an object, grasps it incorrectly, missing or knocking it over:

❶ Use things that are a different colour to the surface of the unit. ❷ Give extra reminders. For example, "The cup is just beside your elbow". ❸ When picking something up use the same hand for guidance - touch the surface the object is on with the side of the hand and slide the hand forward to pick up the object. Or use the other hand as a guide to find where the object is - touch the object with the non dominant or hand he doesn’t use often allowing the reaching hand to use a more precise grip. The brain can note the position of the object through touch not vision. ❹ Practice hand eye co-ordination with games and toys that develop this skill

Q24 Has difficulty seeing something which is pointed out in the distance:

❶ Try zooming in to an item of interest using a digital camera or camera phone. Consider recording the scene so you can talk about it later. Allow your child time to practice using a camera or phone to do this. ❷ Give your child clear directions about where you would like him to look. For example "If you look at the big church, and then look past it you will see your friend." ❸ Play "I spy" games. Encourage your child to chose a distant object and give clues about it so that others have to find and identify the same object. (You could use a video camera or digital camera to help your child find objects in the distance). ❹ Encourage your child to look at the scenery regularly. Ask him to tell you what he sees and talk about it together. ❺ Give your child lots of time to take in what he is seeing. Try not to rush him on to the next thing too quickly. Try to be patient with him ❻ If you spot something your child would enjoy take him to the object so he can see it close up.

Q25 Has difficulty finding a close friend or relative who is standing in a group:

❶ Let your child know who is around. For example, "your friend Jenny is at the gate in a pink jacket." ❷ Make sure friends and teachers say who they are, especially if they meet your child in a busy place. ❸ Point out friends and family in busy places such as the playground. ❹ Encourage your child to listen carefully to voices to help locate friends and family. ❺ Ask your child to call out for person he wants to find. ❻ Ask your child to use mobile phone to call or text the person he wants to find. ❼ Agree a meeting point before hand. ❽ Use your voice to help your child find you. (For example, Mum calls out her child's name so that her voice can be recognised as well as her face (be aware that some children find it hard to work out where a voice is coming from, so waving as well as shouting may help the child identify where Mum is). ❾ Agree a meeting place in advance so your child knows where to find you. ❿ Wear clothing that stands out and is easy to spot, for example a pink jumper or shinny jacket. This item of clothing may then "pop out" at your child and help him to find you. Make sure tell your child about the item of clothing you plan to wear before hand so he knows what to look for. It is also important that the item of clothing can be seen from all angles. i.e. a jumper, not a badge.

Q26 Has difficulty playing team games with a lot of players, especially if there are a lot of players and they are all moving:

❶ Make sure that team members wear shinny/ brightly coloured bibs so that they can be seen more easily. The other team should wear different coloured bibs. ❷ Ask team members to keep shouting out where they are so your child can spot them more easily. ❸ Encourage your child to try sports that involve fewer people and less movement. For example card games or reading.

Q27 Has difficulty finding an item in a supermarket e.g. finding the cereal or magazine:

❶ Give extra spoken help and clear instructions. For example "It is in the middle shelf at the front." ❷ When you visit shops/markets ask your child to find a few things. To start with always ask him to find the same things. Then slowly add in extra items. This may help your child remember where things are and learn how to find his way around the shop. ❸ Make sure your child does not have to look through too many items to find what he is looking for. This can be done by shopping in small shops with small numbers of items on each shelf. ❹ Put only one type of clothing in each drawer or cupboard section. Make sure drawers are not too full. For example, have one drawer or container for socks, one for t-shirts etc.

Q28 Has difficulty locating an item of clothing in a pile of clothes:

❶ Put only one type of clothing in each drawer or cupboard section. Make sure drawers are not too full. For example, have one drawer for socks, one for t-shirts etc. ❷ Try different storage styles and layouts. For example, order clothes across the way or down the way - underwear on the top or far left box, t-shirts in the next box, trousers or skirts in the next and so on. ❸ Hang clothes on a rail or in a wardrobe rather than fold them. ❹ Try hanging clothes in groups, put items of the same type and colour together. i.e. Red tee shirts together, blue tee shirts together, black trousers together. ❺ Only put one type of clothing in each drawer. Don't overfill drawers. i.e. have one drawer for socks, one for t-shirts etc. ❻ Hang all the clothes for one outfit together. ❼ Arrange clothes he will use the night before the night before. ❽ Make sure the walls, carpets and bed covers are plain. This means there is less for your child to 'take in' when he is looking for something. ❾ Use spotlights or a lamp to light up important areas in room i.e. above drawers. ❿ Involve your child in sorting out his belongings, let him decide where things should go.

Q29 Has difficulty selecting a chosen toy in a full toy box:

❶ Store shoes in an elevated shoe rack. ❷ Involve your child in sorting out his belongings. Try not to use too many storage systems. ❸ Put just a few items in each toy box, bag, drawer, cupboard and on each shelf. ❹ Encourage your child to put things back in the same place when he has finished using him. ❺ Label storage boxes and drawers with photos or pictures of what is inside. Try colour or label coded boxes, for example console games in blue boxes, trains in red boxes, dolls in yellow boxes etc. ❻ At school make sure there are only a few things on your child's desk, in his pencil case and in his bag. ❼ Try using 'see through' containers. i.e. pencil case and school bag. ❽ Use clearly labelled storage so that objects can be easily put away after use. This will reduce the number of things your child is trying to see at the same time (visual clutter). ❾ Set up a special area in your child's room for him to put important items i.e. a box or plate on the bedside table for glasses, I pod, mobile phone, TV remote. Encourage your child to always put things back in this place after he has used him. ❿ Have colour or label coded books/ folders for each subject.

Q30 Has difficulty identifying objects when they are on a similar background such as a white tee shirt on a white sheet:

❶ Use different, plain colours and backgrounds.

Q31 Gets lost in places where there is a lot to see, e.g. in a crowded shopping centre or market place:

❶ Make sure you have agreed a meeting point with your child, in case he gets lost while out and about. ❷ Ask your child to stay close to the adult looking after him when they are out and about. ❸ Practice reading and using simple maps and directions with your child. ❹ Give extra practice to help your child find his way around new and unfamiliar places. This may help your child get to know the area. ❺ Help your child to find his way around a new place. You can do this by encouraging your child to go a short distance and then return to you, gradually increase the distance travelled and the number of left / right turns. This could be used at a party venue or supermarket. ❻ Use phonesso that your child can keep in touch, but can also develop his independence.

Q32 Finds copying words or drawings time-consuming and difficult:

❶ Try Voice Activated soft-ware, which will read text and emails to your child. ❷ Reduce the amount of copying your child has to do by putting information on a pre-printed sheet. ❸ Email or scan information to a laptop instead of asking your child to copy it. ❹ Make sure words are clearly visible to your child. The size of print needed will depend on how clearly your child sees (visual acuity) and how well he sees contrast (contrast sensitivity). ❺ Find out what colour of ink and size of print is clearest for your child to see. You should do this for both computer and board work. ❻ Reduce the amount of information on the board. Leave only the most important text. ❼ Make sure the space around the board is clear from anything that could distract from the board itself. i.e. pictures, mobiles. ❽ In class make sure that your child sits face on to the board. Your child should sit at the distance he finds it easiest to read the board. ❾ Make sure the board is well lit. ❿ Let your child to use a mobile phone to photograph writing from the board. This will save him from having to write it down. For example, homework instructions. ⓫ Play games that use your child's ability to remember what he has seen. For example, 'I spy', 'tell me what you saw'. ⓬ Make use of your child's memory for things he hears. For example, speak instructions out loud as well as writing them down. ⓭ Do not ask your child to do more than one thing at a time, for example write and speak or write and listen at the same time. ⓮ Scribe work for your child where possible. This can need a lot of practice, especially if you are scribing for maths, science or languages.

Q33 Has difficulty reading crowded text on paper or on a computer screen, but can cope better if some of the text is covered or removed:

❶ Hide text around the words your child is trying to read using a typoscope (a piece of black card with a viewing slot in it) or a big ruler. This can be moved along each line and from line to line. ❷ Make words (font size) bigger. This will reduce the number of words on the paper or screen at any one time and may make it easier for your child to 'take in' the information. ❸ Make sure your child is not asked to 'take in' too much information at the same time. This can be done by putting only a few things on each page or on the classroom board. ❹ You can try different line spacing, font style and colour contrast. You should be aiming to find the layout that allows your child to read the fastest. Once this layout have been found use it as often as you can. Note you may need books to be scanned and enlarged or converted to double spacing. ❺ Make sure the computer set up is the same every time it is turned on.

Q34 Has difficulty finding letters on keyboard but knows alphabet:

❶ Make key-board keys clearer using brightly coloured alphabet stickers. ❷ Use a larger key-board with bigger keys. ❸ Try using an adapted mouse or keyboard. For example, keyboard with a finger guard or highlighted keys, or a mouse that is enlarged. ❹ Let your child try different computer programmes to reduce the dexterity skills required i.e. clicker 5 clicks on the word, your child will then have to find the word and click with the mouse, not type each individual letter with his fingers. ❺ Try Voice Activated soft-ware. The key-board will then play a less important role in computer work.

Q35 Sits closer to the television than about 30 cm:

❶ See if your child finds it easier to watch TV on a large screen TV. ❷ Make sure there are no objects (visual clutter) between your child's seat and the TV. ❸ Do not put things around the TV. These objects (photos, plants etc) can create 'visual clutter' and increase the amount of information your child is trying to 'take in' at once. ❹ See if your child finds a smaller TV easier to watch. A large screen can make it hard to follow what is happening in all parts of the picture. ❺ Join a film or book club to get older, slower moving films. These usually have fewer special effects.

Q36 Finds it difficult to keep to task for more than 5 minutes, or after being distracted finds it difficult to get back to what they were doing:

❶ Take away unnecessary objects that clutter your child's work area. ❷ If possible, take other distractions away from your child's work area. i.e. sound, movement. ❸ Ask your child to work in short bursts. You can do this by giving tasks in short blocks and breaking down activities. ❹ Time your child's concentration span. Work with your child only for as long as he can concentrate. You may be able to build this up slowly. ❺ Use an alarm clock to set time limits for tasks. This should be based on your child's concentration span. The ❻ Vary tasks and demands. i.e. seating, standing, moving, listening, talking, looking. ❼ Give your child lots of reminders and praise. For example "You are doing well. Just 1 more minute to go". ❽ With your child, make up an unspoken signal that you can give to help your child get back on task. For example, a tap on the shoulder or two claps. ❾ Have a quiet work space for your child at home and in class. Don't have too many things in this space. Too many objects can act as 'visual distractions' and make it harder for your child to find what he is looking for. This work space should not be beside a window. ❿ Let your child have lots of breaks. Let him move around at these times. For instance he could hand out pencils or go for a message. Your child may need to do something active before he gets started on a writing task. ⓫ Make sure your child sits near the front of the class, facing the board and teacher. But make sure your child does not sit at the very front of the class, as he may want to turn around. Sitting near the front of the class may take away 'visual distractions' and the need to look over heads. ⓬ Let your child try using a chair with arms. This may help him to balance. ⓭ Let your child use a fidget object to help him focus on what is being said. This could be "blu tac", a giant paper clip, or a rubber. ⓮ Let your child use head phones or ear plugs so noise does not disturb him.

Q37 Reacts angrily when other restless children cause distraction:

❶ If possible, take other distractions away from your child's work area. i.e. sound, movement. ❷ Let your child use head phones or ear plugs so noise does not disturb him. ❸ See how your child gets on sitting at a separate desk at the end of the group. This may give him more space without leaving him out of the group. If your child has visual attention problems on one side, consider sitting with the affected side next to the wall (i.e. if your child always leaves the food on the right side of his plate or bumps into the right side of a door frame sit him with his right side to the wall).

Q38 Bumps into things when walking and having a conversation:

❶ Let your child know what is coming up. For example, "There is a tree coming up in front of you." ❷ Ask your child to carry out one task at a time. For example, walking or talking or listening.

Q39 Misses objects which are obvious to you because they are different from their background and seem to 'pop out' e.g. a bright ball in the grass:

❶ Do not present too many items at one time. ❷ Space the items out. ❸ Make objects stand out from their background. This can be done by having plain floors, walls and bed spread. ❹ Make sure objects are brightly coloured and a different colour to their background. This can make them jump out'.

Q40 Becomes distressed in places with a lot of clutter or busy environments, such as supermarket or shopping centre:

❶ Do not have too many bits and pieces in areas that your child uses a lot. For example, photos on the wall in class. ❷ Be prepared for difficult behaviour in busy places and take steps to make the situation easier. For example, give lots of advance and warnings, only go to these places for a short time have a reward for afterwards. ❸ Help your child to use his other senses. For example, let him listen to music. ❹ Give your child something to feel. This will use his sense of touch. he could use an object, toy, rubber or giant paper clip. ❺ Give your child something to suck or chew. This will use his sense of taste. You could use chewing gum, mint, lemon or an ice lolly. ❻ Give your child something to smell. This will use his sense of smell. You could use a hanky with some perfume on it. ❼ Ask your child to push the trolley or carry the basket. These activities use deep muscles and can be calming. ❽ Ask your child to help you. For example, when shopping ask him to find things, make sure he has his own job at assembly. ❾ Let your child to take lots of breaks to move around. ❿ Make sure there is a quiet area for your child to use, both at home and at school. This should be a space with very few objects in it. ⓫ Practice in smaller, quieter areas. For example a small dining room, or a small shop rather than the large school dining room or large shopping centre. ⓬ Gradually build up your child's experience of new places. For example, start by taking a trip to a small shop for one item. ⓭ Go to parties early. This will give your child time to get to know the new surroundings.

Q41 Has difficulty recognising close relatives in real life:

❶ Wear clothes that make you easy to spot. For example, a bright pink top or scarf. The item of clothing must be seen from all angles. Tell your child the item of clothing you will be wearing so he knows what to look for. ❷ Teach your child to look for special marks on people that he knows. For example, birthmarks or tattoos. ❸ Practice recognising different voices with your child. ❹ Let your child know that you are there. For example, shout your child's name. ❺ Make sure people know your child finds it hard to recognise faces. Ask people to say hello, and who they are, when they meet your child. ❻ Make sure your child has a relative or friend to help him find people. ❼ Ask your child to shout out the name of the person he is looking for.

Q42 Has difficulty recognising close relatives from photographs:

❶ Use photos of close family and friends to practice spotting people. ❷ Use pictures with only one person to practice identifying people. ❸ Give your child clues. For example, "We saw them here yesterday."

Q43 May mistakenly identify strangers as people known to them:

❶ Give your child extra ideas. For example, "It can't be your Gran because she is at home." ❷ Remind your child to look again.

Q44 Has difficulty understanding the meaning of facial expressions:

❶ Make sure people spending time with your child know it is difficult for him to recognise people. Give extra help and hints. ❷ Find out if your child can tell when you are happy/ sad/ cross etc by the look on your face (facial expression). If he can recognise facial expressions try and work out the easiest distance from him to do this. ❸ Ask your child to listen carefully to the tone of voice, and words being used. This may help him to work out the mood of the person he is talking to. ❹ Practice recognising different facial expressions with your child. i.e. Happy, sad, cross, surprised. ❺ Use words and tone of voice that match the expression on your face. For example "I'm happy, I'm smiling at you". Ask every one spending time with your child to do this. ❻ Exaggerate your voice and use expressed emotions to help your child to understand. For example, by acting very happy, very sad, very angry.

Q45 Has difficulty naming common colours:

❶ Find out if this is due to a problem with colour vision (colour blindness). ❷ Practice the names of the primary colours (red, blue and yellow) using objects that your child knows well.

Q46 Has difficulty naming basic shapes such as squares, triangles and circles:

❶ When learning shape names use 3D models. ❷ Ask your child to touch the shape and say the name. ❸ Practice using 3D shapes. ❹ Ask your child to feel objects with his hands. ❺ Ask your child to make shapes with his fingers in sand or shaving foam. ❻ Make sure your child does not have to look for too many things at the same time. ❼ Encourage your child to play computer games that use recognition skills.

Q47 Has difficulty recognising familiar objects such as the family car, classroom door:

❶ When your child has to find the item that belongs to him from a group of similar items, mark the one that belongs to him with something easy to spot. For example, put a plant on the doorstep of your house so your child knows which house is home, put a hanging toy on the rear windscreen of the car or a cushion on parcel shelf so your child can identify the family car, make sure there is a poster or special mark on his classroom door. ❷ Agree a meeting point in case your child gets lost. ❸ Ask your child to stay close to the adult he is with. ❹ Help your child learn how to find his way around. This can be done by going a little way and then going back to where you started. Gradually build up distance and the number of left / right turns. ❺ Try using a phone. This will let you stay in touch while letting you child get used to finding his way without help.

Q48 Has difficulty navigating familiar environments, e.g. school:

❶ Use brightly coloured tape to mark your child's desk, this may make it easier to find. ❷ If your child gets lost in places he knows (for example, home or school) use a few circle markers, or brightly coloured footprints on the floor, to mark important routes or tell him/her about the directions to followi.e. seat to board, seat to door, hall to bedroom.

Q49 Has difficulty recognising an object if it is partially hidden and not fully visible, or viewed from an unusual angle:

❶ Make sure all items are easy to see and are not on top of each other. ❷ Store items upright. ❸ Try a raised shoe rack. ❹ Make an organised work space for your child. Mark lines on the desk with coloured tape for a book, pencil etc. Don't keep too many things on the desk. ❺ Make sure your child has a schoolbag with a pocket for each item/ group of items. ❻ Try giving your child a flat pencil case with a clearly marked space for each item.

Q50 Has difficulty recognising right and left shoes:

❶ Mark the shoe of your child's dominant side (right if he is right handed, left if he is left handed) with a sticker or pen marker, or decorative bead or initials. This will show which one should go on first.

Q51 Has difficulty recognising people, word, objects if changed in presentation style, i.e. font of text, hairstyle:

❶ Try to approach your child face to face. ❷ Make sure all text given to your child is set out in the same way. i.e The same font, line spacing and colour. ❸ Tell your child if you change the way you look. For example, a new hairstyle.

Q52 Finds it difficult to identify where sound is coming from, i.e. if they shout for you in the house do you have to explain which room you are in:

❶ If your child calls for you, always say what room you are in. ❷ Use actions to show where you are. For example, wave. ❸ Ask your child to send someone, or use a mobile phone to call or text you when he is looking for you.
